# Mapping the Function of Whole‐Brain Projection at the Single Neuron Level

**DOI:** 10.1002/advs.202202553

**Published:** 2022-10-13

**Authors:** Wei Zhou, Shanshan Ke, Wenwei Li, Jing Yuan, Xiangning Li, Rui Jin, Xueyan Jia, Tao Jiang, Zimin Dai, Guannan He, Zhiwei Fang, Liang Shi, Qi Zhang, Hui Gong, Qingming Luo, Wenzhi Sun, Anan Li, Pengcheng Li

**Affiliations:** ^1^ Britton Chance Center and MoE Key Laboratory for Biomedical Photonics Wuhan National Laboratory for Optoelectronics Huazhong University of Science and Technology Wuhan 430074 China; ^2^ Research Unit of Multimodal Cross Scale Neural Signal Detection and Imaging Chinese Academy of Medical Sciences HUST‐Suzhou Institute for Brainsmatics JITRI Suzhou 215100 China; ^3^ Key Laboratory of Biomedical Engineering of Hainan Province, School of Biomedical Engineering Hainan University Haikou 570228 China; ^4^ Chinese Institute for Brain Research Beijing 102206 China; ^5^ School of Basic Medical Sciences Capital Medical University Beijing 100069 China

**Keywords:** whole‐brain projection, two‐photon calcium imaging in vivo, high‐definition fluorescent micro‐optical sectioning tomography (HD‐fMOST), GCaMP6, primary visual cortex

## Abstract

Axonal projection conveys neural information. The divergent and diverse projections of individual neurons imply the complexity of information flow. It is necessary to investigate the relationship between the projection and functional information at the single neuron level for understanding the rules of neural circuit assembly, but a gap remains due to a lack of methods to map the function to whole‐brain projection. Here an approach is developed to bridge two‐photon calcium imaging in vivo with high‐resolution whole‐brain imaging based on sparse labeling with the genetically encoded calcium indicator GCaMP6. Reliable whole‐brain projections are captured by the high‐definition fluorescent micro‐optical sectioning tomography (HD‐fMOST). A cross‐modality cell matching is performed and the functional annotation of whole‐brain projection at the single‐neuron level (FAWPS) is obtained. Applying it to the layer 2/3 (L2/3) neurons in mouse visual cortex, the relationship is investigated between functional preferences and axonal projection features. The functional preference of projection motifs and the correlation between axonal length in MOs and neuronal orientation selectivity, suggest that projection motif‐defined neurons form a functionally specific information flow, and the projection strength in specific targets relates to the information clarity. This pipeline provides a new way to understand the principle of neuronal information transmission.

## Graphical abstract

An approach is established to couple the functional activities with the whole‐brain projection of the same neuron by GCaMP6 sparse labeling and a cross‐modality cell matching. It extends the understanding of structure–function correlations through obtaining functionally relevant projection motifs or projection strengths at the single neuron level.

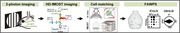

## 1 Introduction

Neuronal projection is the carrier of information flow in the brain. More and more single‐neuron projections were reconstructed to understand the precise framework of information networks,^[^ ^1^, ^2^, ^3^, ^4^ ^]^ and their complexity has been uncovered. Cortical neurons project divergently to multiple regions with nonrandom broadcasting motifs.^[^ ^5^ ^]^ Regardless of whether they are defined as having a cortico‐cortical, cortico‐striatal, cortico‐thalamic, or cortico‐brainstem projection type, their projection patterns at the single‐neuron level remain diverse.^[^ ^3^ ^]^

The relationship between projection and functional information of neuronal populations has been investigated to understand information flow. Targeted areas represent the destination of neuronal information output. The mixed axonal projections of neuronal populations form function‐specific information flows.^[^ ^6^, ^7^, ^8^ ^]^ A comparison of functional information output to different regions suggests that the innervation density contributes to downstream functional specificity.^[^ ^9^ ^]^ Confusingly, the functions of neurons in local areas are heterogeneous,^[^ ^10^, ^11^ ^]^ but they form convergent flows through divergent and diverse projections. It is necessary to know about the relationship between projections and functions at the single‐neuron level.

The relationship between projections and functions was explored in a variety of ways. Axonal calcium imaging,^[^ ^9^ ^]^ special labeling,^[^ ^7^, ^8^ ^]^ and Neuropixels probes^[^ ^12^ ^]^ can be used to record the functions of different targets. In order to clarify the significance of fine connections, studies have combined optical calcium imaging with electrophysiological technology^[^ ^13^ ^]^ or electron microscopy^[^ ^14^, ^15^, ^16^ ^]^ to obtain functional information about synaptic connections in local regions. Recently, a method showed the morphology of neurons using single‐electrode electroporation to label a specific functional neuron.^[^ ^17^ ^]^ Considering a new technology, HD‐fMOST, which has an ≈1–2 order of magnitude improvement in the signal‐to‐background ratios,^[^ ^18^ ^]^ provides an opportunity for genetic calcium probes GCaMP to be used in whole‐brain reconstruction.^[^ ^19^ ^]^ The neurons captured by calcium imaging can be located in the whole brain according to vascular, soma distribution, fiber, or other characteristics.^[^ ^13^, ^14^, ^16^, ^20^, ^21^ ^]^ Our understanding of the relationship between function and projection of individual neurons would be improved by directly bridging the imaging techniques and mapping the complex function to diverse projection at single neuron level.

Here we developed an approach to obtain FAWPS by two‐photon calcium imaging in vivo with HD‐fMOST based on sparse labeling of the genetically encoded calcium indicator GCaMP6 and a cross‐modality cell matching. Applying this approach to the mouse visual cortex, we mapped the FAWPS of L2/3 neurons and investigated the relationship between projection characteristics (projection patterns and strength) and functional preferences.

## 2 Results

### 2.1 Achieving the FAWPS

**Figure 1:**
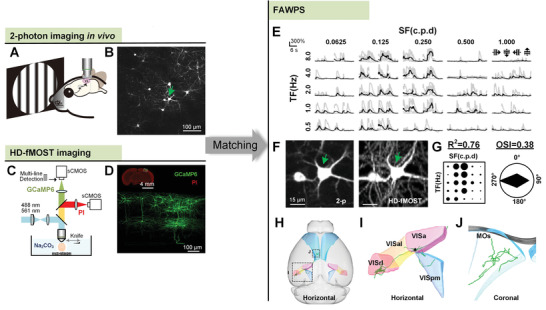
Mapping the FAWPS. A) Two‐photon imaging in vivo of awake mice with visual stimulation for recording the neuronal activity. B) Two‐photon calcium imaging by sparse labeling with GCaMP6. Scale bar, 100 µm. C) The HD‐fMOST system for whole‐brain imaging. D) Top: Merged maximal‐intensity projection of coronal section of HD‐fMOST images. The projections are 100 µm for GCaMP6 and 5 µm for PI. Scale bar, 4 mm. Bottom: zoomed‐in section of GCaMP channel in the upper image. Scale bar, 100 µm. Images in B,D) come from the same brain. E–J) Function and projection of the same neuron indicated by the green arrow in F), hereinafter referred to as Demo Neuron. E) Calcium curves of Demo Neuron in response to three‐factor visual stimulation. Black lines represent the average Δ*F*/*F* for five trials (gray lines). F) The matched Demo Neuron in two‐photon imaging in vivo (left) and HD‐fMOST (right). Scale bar, 15 µm. G) Quantification of the visual function characteristics of Demo Neuron. Left: *R* ^2^ represents the spatiotemporal frequency preference. SF, spatial frequency; TF, temporal frequency. The radius of each circle is proportional to max Δ*F*/*F*. Right: OSI represents the orientation preference, and polar plot shows the max Δ*F*/*F* for different motion directions. H) Whole‐brain projection of Demo Neuron. Colors mark different target areas, and the detailed magnifications of the dashed box sections are presented in I) and J). The soma is shown as a black triangle in I). VISa, anterior area; VISal, anterolateral visual area; VISrl, rostrolateral visual area; VISpm, posteromedial visual area; MOs, secondary motor area.

**Figure 2:**
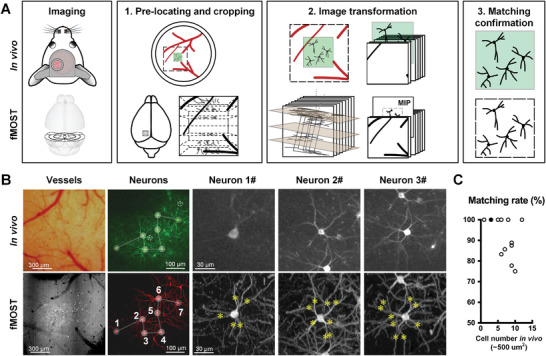
Cell matching between two‐photon imaging in vivo and HD‐fMOST. A) Schematic of cross‐modality cell matching. Left: the horizontal view of a local cortical area within the cranial window during two‐photon imaging (top), and the continuous coronal view of whole‐brain imaging (bottom). Right three figures show the steps for cell matching. Step 1, dashed box (top) indicates the region in the whole‐brain image (bottom) corresponding to the calcium imaging area, which will be cropped out. Step 2, rigid transformation (left) and 2D affine transformation (right) to make the perspectives similar. Step 3, matching cells according to the distribution of somas and fibers. Green pictures represent the in vivo images. B) Example of cell matching between images from two‐photon imaging and HD‐fMOST. Upper left: Blood vessels in the cranial window captured before calcium imaging with a dissection microscope. Bottom left: Blood vessels reconstructed from the intensity‐projection of images from HD‐fMOST after a rigid transformation. Scale bar, 300 µm. Broken lines indicate the distribution pattern of neurons (scale bar, 100 µm), and yellow asterisks identify the similar branch features (scale bar, 30 µm). Dotted circles indicate the somas that failed to be matched. C) Matching rate showed as a function of the labeled cell numbers in vivo. The solid black dot represents *n* = 3. Average matching rate = 93% ± 3% (mean ± SEM, 14 images from 7 brains).

To couple neuronal function with whole‐brain axonal projection, we bridged two‐photon calcium imaging in vivo with high‐resolution whole‐brain imaging of the same mouse brain through labeling with the genetically encoded calcium indicator GCaMP6^[^ ^22^, ^23^ ^]^ (**Figure** **1**). First, a mixture of rAAV viruses containing low‐titer hSyn‐Cre and high‐titer Cre‐dependent reporter hSyn‐DIO‐GCaMP6 was injected to implement sparse labeling in mouse visual cortex (Figure 1B,D, from the same brain). Then, using motion vision as a model, the neuronal activities in L2/3 evoked by moving grating were recorded in awake mice (Figure 1A). In order to describe the functional response characteristics of neurons, we used a three‐factor, multigradient stimulus (including 5 temporal frequencies, 5 spatial frequencies, and 4 directions) and plotted the calcium curves of neurons responding to these 100 kinds of stimuli. The goodness (*R* ^2^) of a 2D Gaussian fit was used to quantify the spatiotemporal preference, and orientation selectivity index (OSI) was used to characterize the orientation preference (details see the Experimental Section). Next, the brain was embedded in resin and immersed in an alkaline buffer^[^ ^19^, ^24^ ^]^ for high‐resolution whole‐brain imaging with an HD‐fMOST system^[^ ^18^ ^]^ (Figure 1C). This system included two separate detection channels for imaging GCaMP6‐labeled neuronal projections and PI‐stained cellular nuclei simultaneously to facilitate precise superimposition of the long‐range projections and anatomical annotation. Afterward, the precise positioning of GCaMP6‐imaged neurons in the whole brain was determined by cell matching (Figures 1F and 2). Finally, the functional annotations of axonal projections were obtained after axonal reconstruction of matched neurons (Figure S1A,B, Supporting Information).

Neurons were registered with the anatomic reference atlas^[^ ^25^ ^]^ using cyto‐architecture obtained from the PI channel of the HD‐fMOST system (Figure S2, Supporting Information). As shown in Figure 1, a demonstration neuron had a strong spatiotemporal and moderate orientation preference (Figure 1G, *R* ^2^ = 0.76, OSI = 0.38), and projected to the anterior area (VISa), anterolateral visual area (VISal), rostrolateral visual area (VISrl), posteromedial visual area (VISpm), and secondary motor area (MOs) (Figure 1H–J). The signal‐to‐noise ratio (SNR) at different points along intact axons was also measured (Figure S1C, median SNR = 24.8, Supporting Information), and all axon endings with their SNRs are displayed in Figure S1D,E (Supporting Information) (median SNR = 29.0). These results suggest the feasibility of FAWPS compared to what has been reported for axonal tracing.^[^ ^1^, ^26^, ^27^ ^]^

Besides, we applied this strategy to the acquisition of cell‐type FAWPS. Testing with rAAV‐SST‐Cre virus showed the visual response as well as dendritic and axonal morphology of one SST neuron (Figure S3, Supporting Information).

### 2.2 Cell Matching Between Two‐Photon Imaging and Whole‐Brain Imaging

Cell matching of cross‐modality data is one of the requirements for integrating the function and structure of a neuron. Two‐photon microscopy captures 2D calcium images in the horizontal plane over a region of ≈500 µm^2^, while the HD‐fMOST system scans the coronal plane to obtain a 3D image of the whole brain on the centimeter scale (**Figure** **2**A, left). Thus, cell matching of cross‐modality data needs to overcome the differences in both the scope and the viewing angle. Our workflow to achieve this included cropping, image transformation and matching confirmation steps. The visual cortex was targeted, and an image stack around the injection site was cropped out from the whole‐brain datasets (Figure 2A‐step 1). Then, a rotational rigid transformation was carried out on the image stack to match the normal vector of the cortex contour with the *z*‐axis of the in vivo imaging (Figure 2A‐step 2). The blood vessels on the cortical surface are shown against the fluorescence background with HD‐fMOST. Based on the vascular morphologies in the cranial window and the depth during two‐photon imaging, the approximate region of the neuronal population was confirmed. HD‐fMOST images of an appropriate thickness were used to produce a maximal‐intensity projection (MIP). At least three landmarks (such as the somas near the blood vessels or distinct branching and branch points) were identified for an affine transformation^[^ ^13^, ^16^ ^]^ between the two‐photon images and MIP. The neurons were then explicitly matched according to the furcation of fibers and the relative positions of somas at the same time (Figure 2A‐step 3,B; and Figure S4A, Supporting Information). Finally, the coordinates of functionally determined neurons in the original whole‐brain datasets were obtained through an inverse transformation.

To evaluate the matching strategy, we counted the matching rate and the density of labeled neurons. The matching rate reached 93% ± 3% (mean ± SEM) when ≈3–9 neurons (≈25–75% percentile, in ≈500 µm^2^) were labeled in vivo (Figure 2C), where the matching rate was defined as the percentage of the successfully matched cells in visually responsive cells. The main reason for matching failure was the absence of fiber morphology in the in vivo images, which may result from the defocus or weak fluorescence of the proximal fibers of the soma. There was no strong correlation between the labeling density and matching rate (Spearman *r* = −0.55, Figure S5A,D, Supporting Information). This indicates that the matching strategy is not affected by the density of sparse labeling.

### 2.3 Quality Control of Brain‐Wide Axonal Reconstruction

**Figure 3:**
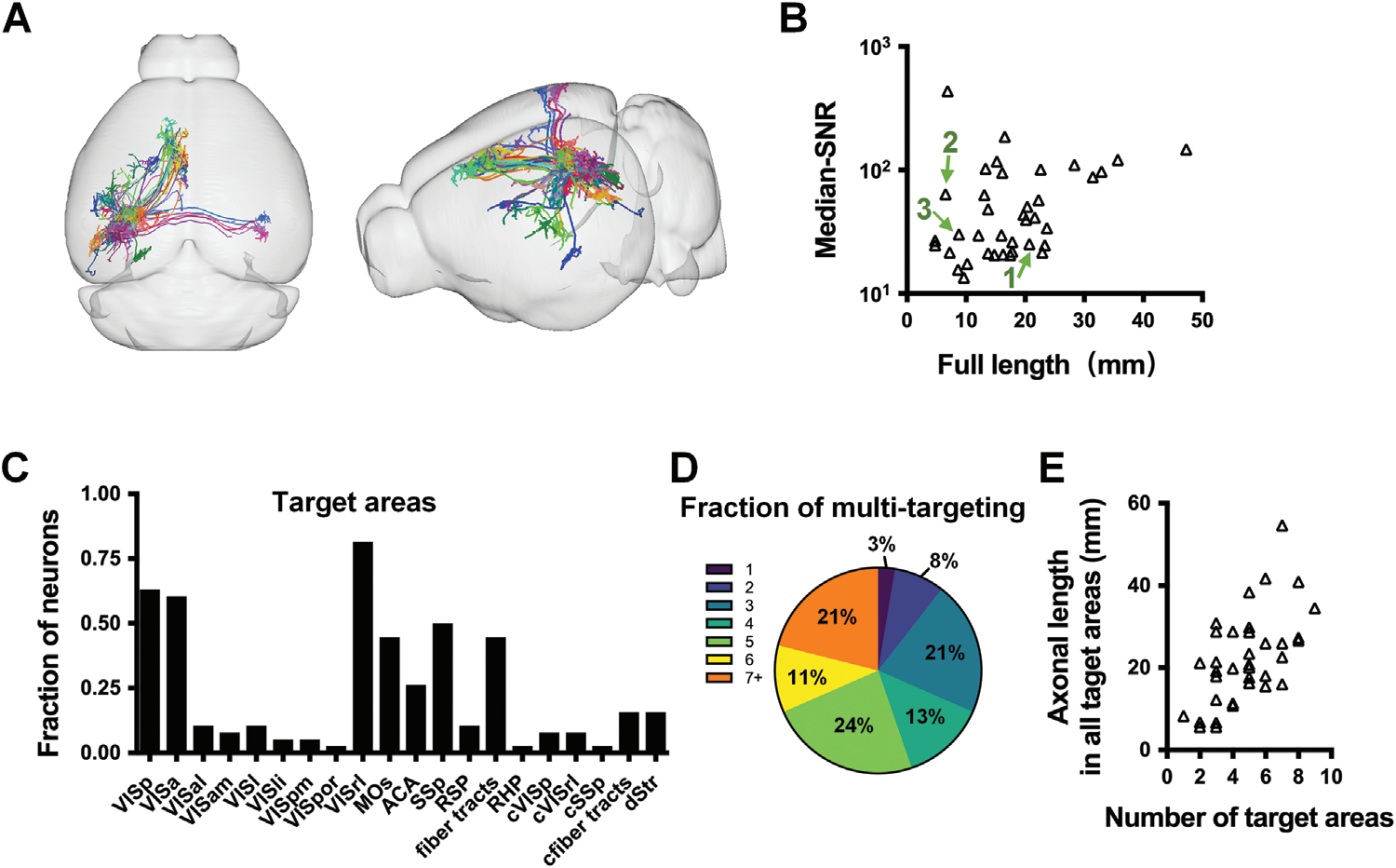
Reconstruction of axons labeled by GCaMP6. A) Overlay of all traced single neurons in Allen Reference Atlas space, *n* = 38 neurons. Left, horizontal view; right, oblique side view. B) Scatter plots of the median values of full‐length (raw length before registration) SNR for 38 neurons. The green arrows indicate the neurons showed in Figure S9 (Supporting Information). C) The fractions of neurons projecting to each of the 20 target areas. VISp, primary visual area; VISa, anterior area; VISal, anterolateral visual area; VISam, anteromedial visual area; VISl, lateral visual area; VISli, laterointermediate area; VISpm, posteromedial visual area; VISpor, postrhinal area; VISrl, rostrolateral visual area; SSp, primary somatosensory area; RSP, retrosplenial area; RFP, retrohippocampal region; cVISp, contralateral primary visual area; cVISrl, contralateral rostrolateral visual area; cSSp, contralateral primary somatosensory area; cfiber tracts, contralateral fiber tracts; dStr, striatum dorsal region. D) Percentage of multitarget neurons. E) Axonal length in all target areas plotted as a function of the number of target areas. The areas that received at least 1 mm of axonal innervation were considered to be targets. Median total length: 21.4 mm, quartile range: 14.7 ≈28.7 mm; Average target number: 4.8 ± 2.0 (mean ± S.D.); Average length per target area: 4.9 ± 2.3 mm (mean ± S.D.).

Overall, we reconstructed in 38 out of the 72 matched neurons (**Figure** **3**A). The success rate of axonal tracing was 61% ± 8% (mean ± SEM), which was influenced by the labeling density (Spearman *r* = −0.62) (Figure S5B–D, Supporting Information). The neurons were excluded either because the main branches were entangled, which was negatively correlated with the tracing rate (Spearman *r* = −0.88, Figure S5E,F, Supporting Information), or the signal was too weak, which included the neurons whose primary axons were difficult to distinguish from background or with unbranched axons in all target areas.

The reliability of GCaMP for whole‐brain axonal reconstruction is a cornerstone of FAWPS, which had been verified in three aspects: axonal labeling, reconstructed signal quality, and projection pattern.

To illustrate the integrity of GCaMP6 labeling, GCaMP6‐labeled axons were compared with the membrane protein‐labeled ones. A red membrane‐localized fluorescent protein, mRuby3.Membr, was coinjected with GCaMP6 into VISp, and 2 weeks later their signals were collected by confocal imaging on brain slices. For GCaMP6 signal enhancement, an alkaline buffer was used during imaging, considering that GCaMP is pH sensitive.^[^ ^22^ ^]^ Here, the effect of alkaline buffer on the signal of two fluorescent proteins was first checked. The signal‐to‐back ratio (SBR) in axons labeled by GCaMP6 was 3 times higher than that before (before, 1.68 ± 0.32; after, 5.23 ± 0.82. mean ± SEM, *p* < 0.0001), but the SBR of the red fluorescent protein did not change significantly (before, 4.07 ± 0.72; after, 3.27 ± 0.59. mean ± SEM, *p* = 0.0637) (Figure S6, Supporting Information). Then, the colabeling of axons was examined in brain wide. Most projections of VISp neurons were colabeled in the telencephalic and subcortical areas, including MOs, Anterior cingulate area (ACA), dorsal‐Striatum (dStr), Thalamus (TH), Superior Colliculus (SC), and Pons (Figure S7, Supporting Information). Moreover, the varicosities (putative boutons) in TH, SC, and Pons were also filled with GCaMP6. The colabeling ratios of axonal segments were calculated and they were similar in different target areas, regardless of the distance of the axon path to the target region (Figure S8, Supporting Information). The average colabeling ratio reached 94.5% ± 1.5% (mean ± SD). These results showed the labeling integrity of GCaMP6 for axons that comparable to membrane marker and suggested the feasibility of GCaMP6 for whole‐brain axonal reconstruction.

To verify the reconstructed signal quality with GCaMP6 labeling, the SNRs of intact axons of each neuron were calculated and compared with GFP, which has been widely used for axonal tracing. The median SNR of full‐length axons here ranged from 13.5 to 434.1 (Figure 3B, 64.5 ± 12.1, mean ± SEM), which was comparable to 15.0–18.0 of GFP.^[^ ^1^, ^26^, ^27^ ^]^ Correlation analysis showed that the reconstruction length was not correlated with the median SNR (Spearman *r* = 0.35). The SNRs of intact axons of several neurons were displayed in Figure S9 (Supporting Information). The data showed that SNR values remained stable from the proximal to the distal of the axon, regardless of the axonal length or distance between the soma and the axon terminal. This suggests that the traced axonal length is independent of the SNR value.

All reconstructed neurons displayed typical intratelencephalic (IT) projection. Their axons generally targeted 20 areas (each target area was defined by having a total axon length >1 mm^2,5^), including 14 ipsilateral cortex areas, 3 contralateral cortex areas, dorsal striatum (dStr) in the ipsilateral hemisphere, and bilateral fiber tracts (Figure 3C). The 7 areas with projections from more than 25% of neurons were VISrl, primary visual area (VISp), VISa, primary somatosensory area (SSp), fiber tracts, MOs and ACA. Of all the neurons analyzed, 97% projected to more than one area, and 21% targeted at least seven areas (Figure 3D). The axonal lengths in all target areas were plotted as a function of the number of target areas (Figure 3E). The average number of target areas was 4.8 ± 2.0 (mean ± SD), and the average length per target area was 4.9 ± 2.3 mm (mean ± SD).

### 2.4 Functional Annotation to Projection Patterns

Whole‐brain projection provides the details including all projection areas and the projection strength in target areas, which have different meanings for the information transmission.^[^ ^9^, ^20^ ^]^ The projection features of these two aspects were investigated separately.

**Figure 4:**
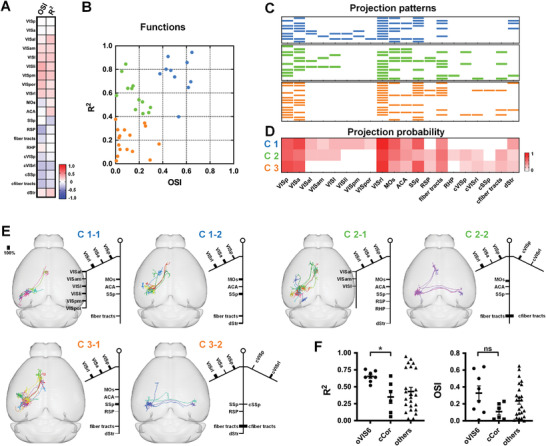
The functional features of projection areas. A) Spearman correlation analysis between the preference index (*R* ^2^ and OSI) and projection areas (with no strength). Colors encode the degree of correlation. B) K‐means clustering of neurons according to *R* ^2^ and OSI. *n* = 38 neurons. C) The whole‐brain projection patterns of individual neurons in each class. Blue indicates the Class 1 (C1), green indicates the class 2 (C2), and orange indicates the Class 3 (C3). D) The probability of projection areas for each neuronal class. E) Finer‐scale structural motifs within three neuronal classes. In each motif: left, the reconstructed axons in brain; right, projection motifs. The thickness of short lines represents the probability of projection. F) Functional preference of special motifs. oVIS6: a motif that includes VISal, VISam, VISl, VISli, VISpm, and VISpor. cCor: contralateral cortical projection motif. Error bars, ± SEM. Mann–Whitney test, two‐tailed. *p  <  0.05; ns, nonsignificant.

Spearman correlation analysis showed that there was no significant correlation between single target areas and functional preference (**Figure** **4**A). Single‐neuron axons target multiple areas in a variety of patterns, and the patterns were investigated according to the functional preferences.

The neuronal functions in visual cortex are diverse in orientation and spatiotemporal preferences.^[^ ^6^, ^7^, ^8^, ^9^, ^11^ ^]^ Here, neurons were divided into three classes using the unsupervised clustering to show their different preference intensity in two functional responses: high spatiotemporal & high orientation preference (C1), high spatiotemporal & low orientation preference (C2), and low spatiotemporal & low orientation preference (C3) (Figure 4B, there was no neurons with low spatiotemporal & high orientation preference in our data). All neurons displayed different projection patterns in terms of area identity (Figure 4C), but the projection probabilities among three neuronal classes were different in some special areas (Figure 4D). The three classes of neurons had high probability projection (more than 50%) in VISp, VISa, and VISrl, and low probability projection (less than 50%) in ACA and Str. C1 neurons had abundant projection in the ipsilateral cortex, not only in the presence of more neurons projecting to MOs (60%) and SSp (60%), but also in the presence of extensive projection of higher visual areas (HVAs). C1 covered other six higher visual areas (oVIS6): VISal, VISam, VISl, VISli, VISpm, VISpor (*n* = 4/10 neurons), C2 covered three (*n* = 4/12 neurons), and C3 covered none. Unlike C1, C2, and C3 have contralateral cortical (cCor) projections (C2, *n* = 3/12 neurons, C3, *n* = 3/16 neurons), and RSP projection (C2, *n* = 2/12 neurons, C3, *n* = 2/16 neurons). Between C2 and C3, C2 has a high probability of MOs projection (50%) and C3 has a high probability of SSp projection (63%). The difference in oVIS6, RSP, and cCor projection subsets suggested the different projection patterns may derive from definable motifs on a finer scale^[^ ^3^ ^]^ among three functional populations.

The projection motifs in functional classes were further examined (Figure 4E) in terms of cCor projection and oVIS6 projection. Two motifs were observed in C1: C1‐1 could selectively project to ≈2–3 areas in the oVIS6, and C1‐2 could project to dStr. Both C2 and C3 can be separated by the presence or absence of contralateral projections. Compared with the contralateral projection neurons in C2 (C2‐2), ipsilateral projection neurons were not only likely to project to dStr, RSP, and RHP, but also can selectively project to 1 or 3 areas in the oVIS6 (C2‐1). Both C3‐1 and C3‐2 may project to dStr, different from the difference between ipsilateral and contralateral projection in C2. Besides, MOs and ACA projections only appeared in ipsilateral projection neurons C3‐1. It was noted that the oVIS6 motif and cCor motif repeatedly occurred in the classes with high spatiotemporal preference (C1 and C2) and the classes with low OSI (C2 and C3), respectively. Statistical analysis showed that oVIS6 motifs has a stronger spatiotemporal preference compared to the contralateral motif (Figure 4F, *p* = 0.0176). It indicated that these two motifs may underlying different functional preference.

### 2.5 Functional Annotation to Projection Strength

**Figure 5:**
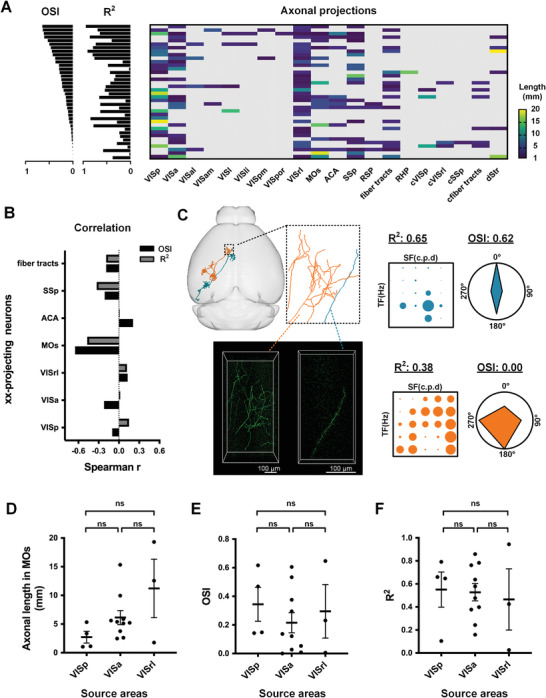
Functional annotation related to the projection strength in the MOs. A) FAWPS of L2/3 neurons in visual cortex. Rows show individual neurons, *n* = 38. Neurons are sorted from high to low by OSI. The colors in heatmap reflect the axonal length in each targeted area. B) Spearman correlation coefficient between functional preference indices (OSI and *R* ^2^) and the axonal length in the specific areas of six kinds of targeted neurons. C) FAWPS of the neurons with a simple axon (blue) or complex axons (orange) targeted in MOs. Top: Registered projection in the standard brain space (left) and axons in MOs (right). Bottom: the axons in MOs from raw brain. Scale bars, 100 µm. D–F) Statistical difference in axonal length in MOs, OSI, and *R* ^2^ between different source neurons. Error bars, ± SEM. Kruskal–Wallis test with Dunn's post hoc test. ns, nonsignificant.

Furthermore, the relationship between projection strength and functional preferences in a single target area were determined. Whole‐brain axonal reconstruction of functional neurons provide insight into the innervation density by measuring axonal length in the target areas (**Figure** **5**A). We focused on the seven types of projecting neurons with a proportion of more than a quarter (Figure 5B, VISp‐, VISa‐, VISrl‐, MOs‐, ACA‐, SSp‐, and fiber tracts‐projecting neurons). Correlation analysis indicated that only the axonal length in MOs was significantly correlated with OSI (*r* = −0.652, *p* = 0.006). The neurons projected to MOs with shorter axons had higher orientation selectivity (Figure 5C). Besides, OSI and *R* ^2^ of MOs‐projecting neurons were correlated (Figure S10, *r* = 0.63, *p* = 0.008, Supporting Information), which meant the neurons with high OSI were also more likely to have high *R* ^2^. In addition, the somas of MOs‐projecting neurons were located in three visual subregions according to the Allen Common Coordinate Framework: VISp, VISa, and VISrl. However, there were no significant differences in axonal length and preference intensity among them (Figure 5D–F). These results suggested that visual neurons may transmit clear motion vision signals to the MOs through concise axonal projection, and it was independent of soma position.

## 3 Discussion

With the sparse labeling based on GCaMP6, functional imaging in vivo was directly combined with the whole brain structural imaging to mapping FAWPS. Compared to using chemical calcium dyes and cell‐by‐cell targeted injection,^[^ ^17^ ^]^ genetic encoded calcium probes could capture the multilevel functional response and diverse whole‐brain projections in a noninvasive way. Our approach can also target different cell types using genetic strategies, such as the SST neurons. With the development of transcriptomics, the fusion with transcriptome is possible through molecularly‐defined markers.^[^ ^28^, ^29^ ^]^

It is a universal pipeline that is easily accessible. With the development of technologies such as labeling and imaging, the efficiency of FAWPS can be further improved. New genetically encoded calcium indicators provide better calcium‐sensitivity to enhance functional imaging efficiency, like the jGCaMP7^30^ and jGCaMP8.^[^ ^31^ ^]^ For example, jGCaMP7b has a brighter fluorescent baseline that facilitates imaging in vivo of neurites and neuropil. And the sampling rate can also be boosted by the efficiency of in vivo imaging, matching as well as tracing. Recently, large‐field^[^ ^32^, ^33^ ^]^ and volumetric two‐photon imaging^[^ ^34^, ^35^ ^]^ have increased the throughput of neuronal activity recording, machine learning has involved in batch matching task,^[^ ^21^ ^]^ and artificial intelligence (AI) has contributed to tracing.^[^ ^36^, ^37^ ^]^ These technological advances should improve the throughput of FAWPS in future.

The accuracy of mapping FAWPS depends on reliable axonal reconstruction and cell matching. The reliability of axonal reconstruction based on GCaMP6 labeling was evaluated in comparison to that of GFP, which has been widely used for labeling axonal projection. Alkaline activation was used to enhance the fluorescence signal^[^ ^19^, ^22^, ^24^ ^]^ and HD‐fMOST can inhibit background noise, thus improving the SNR of GCaMP6 in whole‐brain imaging compared with GFP. The number of target areas and average axonal length of a single target area were measured, and were also found to be similar to the projections labeled by GFP.^[^ ^2^, ^5^ ^]^ For the L2/3 IT neurons, reconstructed neurons here showed an average number of target areas similar to that of GFP^[^ ^2^ ^]^ (4.7 in our study vs 5.4). Their total axonal length (median, 21.4 mm) was longer than VISp neurons^[^ ^5^ ^]^ but shorter than SSp, SSs, Mop, and SSs neurons.^[^ ^2^ ^]^ This may be due to the different source areas and the different proportions of contralateral projection neurons. VISp neurons were then selected and their average axonal length for a single target area reached 5.3 ± 2.5 mm (Figure S11F, mean ± SD, Supporting Information), similar to the L2/3 VISp single‐neuron reconstruction reported by Han et al.^[^ ^5^ ^]^ (4.6 ± 2.2 mm). We conclude that GCaMP6 can be used for whole‐brain axonal tracing, and it provides a direct way to combine neuronal activity and projection at the single‐neuron level.

Accurate cell matching is the cornerstone of reliable FAWPS data analysis. The fibers captured by calcium imaging in vivo are interleaved with each other, with fragmented and blurred morphology (Figure 2B; and Figure S3, Supporting Information), resulting difficulty in matching. In contrast to previous publications^[^ ^13^, ^14^, ^16^, ^20^, ^21^ ^]^ which mainly relied on relative soma position, we used a dual criterion: soma position and fiber morphology. Manual matching with this strict criterion provided an accurate linkage between function and structure.^[^ ^16^ ^]^ Additionally, soma location and axonal projection of matched neurons can be precisely registered in the intact brain with an fMOST self‐calibrating channel, instead of being located in part of the brain.^[^ ^16^ ^]^ This matching process performed in whole‐brain datasets also enabled us to map FAWPS without auxiliary labeling (blood vessels, astrocytes, etc.).^[^ ^13^, ^14^, ^16^, ^20^, ^21^ ^]^

The reconstructed neurons targeted multiple areas in highly variable patterns. Han et al.^[^ ^5^ ^]^ found that single neurons project broadly in nonrandom motifs, and our results displayed a specific case of nonrandomness: function‐related projection motifs. Although the functional projection of populations indicate that different targets have different preference characteristics,^[^ ^7^, ^8^, ^38^ ^]^ the divergence and diversity of whole‐brain projection patterns of individual neurons remind that the information flows are more than one‐to‐one correspondence. We uncovered an oVIS6 projection motif from the whole‐brain projections, which had a stronger spatiotemporal frequency preference than the cCor projections. The oVIS6 areas are all part of HVAs, which has traditionally been considered for further processing of visual information. The functional projection from L2/3 VISp to HVAs show that the functional specificity of targets is determined by different signal proportion,^[^ ^9^ ^]^ and our findings imply that there is a high probability of information with spatiotemporal frequency preference in a motif consisted of some higher visual areas.

According to the Allen Common Coordinate Framework, our neurons were located in four visual subregions, but the source areas had no effect on neuronal functional preferences (Figure S11, Supporting Information). Neurons with each source area had the highest projection probability in their native area (12/12 for VISp neurons, 5/6 for VISrl neurons, and 10/10 for VISa neurons), except for VISam neurons (1/3 neurons). Interestingly, VISrl is a high‐probability projection region for the other three source neurons (11/12 VISp neurons, 3/3 VISam neurons, and 5/10 VISa neurons). oVIS6 projecting neurons are scattered across three source regions (2/12 in VISp neurons, 2/3 in VISam neurons, and 2/10 in VISa neurons). Neurons projected to high probability areas have various functional preference intensities, while low probability areas are more likely to represent a particular projection motif with functional specificity.

Here we found that the innervation density of visual neurons to MOs was related to the intensity of orientation selectivity. Functionally, appropriate behavior requires constant reference to visual information.^[^ ^38^, ^39^ ^]^ Orientation detection are the primary functions of motion vision, and their direct transmission meets the needs of dynamic behavior adjustment. Visual neurons deliver clearer visual motion characteristics to MOs with simple axons, which are thought to carry primary information and participate in behavioral information integration using concise resources. This is more conducive to the efficiency of the neural network.

Due to the diverse and divergent projections, more studies are still needed to clarify the principles of FAWPS. The FAWPS of L2/3 visual neurons suggest that primary visual motion information delivery may be through a selection of low‐probability projection motif or simple innervation in specific target (MOs). Other high‐probability projections and complex innervation may be associated with higher‐level or highly mixed information transmission. In addition, the interpretation of FAWPS also needs to consider the neuronal types^[^ ^40^, ^41^, ^42^ ^]^ and the functional model applied. Many other functions involving visual neurons have been reported, such as shape,^[^ ^43^, ^44^ ^]^ color,^[^ ^45^ ^]^ and navigation,^[^ ^46^, ^47^ ^]^ and there may be more than one functional representation for a given neuron.^[^ ^48^ ^]^ Broader information flows may be found through mapping FAWPS with different labeling strategies and functional models.

## 4 Experimental Section

### Animals

Male C57/B6J mice were obtained from Beijing Vital River Laboratory Animal Technology Co., Ltd. Mice were maintained under conditions of 22 ± 1 °C and 55 ± 5% humidity with a 12 h light/dark photoperiod (light on at 7:00) and were provided food and water ad libitum. All animal experiments were conducted in accordance with the Guidelines of the Animal Care Facility of Huazhong University of Science and Technology (HUST) and were approved by the Animal Ethics Committee of HUST (No. S613).

### Virus

The AAV2/9‐hSyn‐Cre‐WPRE‐pA (1× 10^13^ V.G. mL^−1^), AAV2/9‐Ef1*α*‐DIO‐GCaMP6m‐WPRE‐pA (1× 10^13^ V.G. mL^−1^), AAV2/9‐hSyn‐DIO‐GCaMP6m‐WPRE‐pA (1× 10^13^ V.G. mL^−1^), and AAV2/9‐hSyn‐DIO‐mRuby3.Membr (1× 10^13^ V.G. mL^−1^) were purchased from Shanghai Taitool Bioscience Co.Ltd., Shanghai, China. A mixture of high‐titer GCaMP6 virus and low‐titer Cre virus can reduce the density of labeled neurons without compromising the expression level.^[^ ^26^, ^49^ ^]^ 0.01 m phosphate buffered saline (PBS, Sigma‐Aldrich Inc., St Louis, MO) was used to dilute the Cre virus. In the sparse labeling, the concentration ratio of AAV‐hSyn‐Cre to AAV‐DIO‐ GCaMP6m was 1: 1000 or 1: 10 000. In the dual‐color labeling, the concentration ratio of AAV‐hSyn‐Cre to AAV‐DIO‐ GCaMP6m to AAV‐DIO‐mRuby3.Membr was 1: 100: 100.

### Viral Injection and Cranial Window Implant

Mice (6–8 weeks old, male) were anesthetized with an intraperitoneal injection of 2% chloral hydrate (0.10 mL/10 g) and 0.015% xylazine (0.08 mL/10 g) before surgery and then placed in a mouse stereotaxic instrument. With aseptic surgical procedures, a circular craniotomy (≈3.5 mm in diameter) was performed over the visual cortex. Viral injection was performed using a glass pipette beveled at 30° with a 15–20 µm opening and back‐filled with mineral oil. A hydraulic manipulator (Narashige, MO10) was used to load and inject the viral solution (20 nL). To prevent backflow during withdrawal, the pipette was kept in the brain for over 5 min. The single injection site in the left hemisphere was centered at 3.2 mm posterior to Bregma, 2.6 mm lateral from midline, 0.45 mm below pia. Two injection sites were ≈500 µm apart. The glass window was a piece of coverslips (Fisher Scientific No. 1.5, machined into a circle with a diameter of 3.5 mm) glued under a titanium ring (outer diameter 4.5 mm, inner diameter 3.0 mm). The glass was embedded into the opening by sealing the titanium ring. A custom titanium head‐post was attached to the skull with cyanoacrylate glue and dental acrylic.

### Visual Stimulation

Visual stimuli were presented by flat panel monitor (Dell, S2417DG, resolution 2560*1440, refresh rate 165 Hz). The screen was positioned 15 cm from the right eye, and oriented at ≈30° to the long axis of the animal. The area of visual stimulation was a 30 cm diameter circle, which covered the visual field at 90° × 52°.

The moving grating was generated by Psychopy 3.0, with a period of 6 s movement and periods of 3 s rest before and after. In order to activate as many neurons as possible, a matrix stimulation strategy, in which each cell was tested for orientation and temporal and spatial frequency tuning was designed. 5 temporal frequencies (0.5, 1.0, 2.0, 4.0, and 8.0 Hz) and 5 spatial frequencies 5 spatial frequencies (0.0625, 0.125, 0.25, 0.5, and 1.0 cycles per degree) were combined into 25 units. In each unit, the grating moved randomly in one of 4 directions (90°, 180°, 270°, and 360°), and each direction was repeated 5 times. The actual sequence of movement was recorded for the segmentation and rearrangement of two‐photon images.

### Two‐Photon Imaging

In vivo imaging was performed with a two‐photon fluorescence microscope (Olympus FV3000, 25x /1.05NA) 3 weeks after viral injection. Mice were head‐fixed and awake during the imaging period. Before the formal experiment, they were given adaptive training (at least three times, increasing from 0.5 to 2 h) to reduce struggling and prevent large body movements such as running. Each experiment lasted ≈2–3 h, and multiple sections could be imaged within the same mouse, but not on the same day. GCaMP6 was excited at 920 nm with a Mai Tai HP DeepSee laser. Emitted fluorescence photons were detected by a GaAsP‐NDD PMT. High‐resolution Galvano Scan (512 × 512, 1.0 F s^−1^) was used to record the vascular information and neuronal depth during 3D imaging. High‐speed Resonant Scan (512 × 512, 30 F s^−1^) was used to perform functional recoding (roundtrip, average 2, 15 F s^−1^).

### Two‐Photon Image Processing and Analysis

The time‐lapse calcium imaging stacks were analyzed with custom programs written in MATLAB (versionA 2017a, MathWorks) referred by Sun et al.^[^ ^50^ ^]^ Briefly, raw data were corrected using a cross‐correlation‐based registration algorithm. This registration was iterating using a template obtained from an average projection of one‐third of the stack. The template was also the functional image used in cross‐modality cell matching. Somas were outlined by hand as regions of interest (ROIs). For each ROI, the average fluorescence intensity in the resting period as the baseline fluorescence *F*_0_ was used, and its calcium transient as Δ*F*/*F* (%) = (*F*−*F*_0_)/*F*_0_ × 100% was calculated. A neuron was considered responsive if its max Δ*F*/*F* during the presentation of visual stimuli was above 10% and repeated at least twice. The final calcium transient to each visual stimulus was the average of five trials.

For orientation preference, the max‐Δ*F*/*F* of all SF and TF were averaged to obtain a set of responses to the four directions. The responses to 180° and 360° were averaged as the horizontal orientation responses, and those to 90° and 270° were averaged as the vertical orientation responses. The orientation selectivity index (OSI) was used to quantify the strength of orientation preference as follows

(1) $$OSI=\frac{\left(R_{\mathrm{pref}}-R_{\mathrm{ortho}}\right)}{\left(R_{\mathrm{pref}}+R_{\mathrm{ortho}}\right)}$$

To evaluate the spatiotemporal frequency preference, the peak of max Δ*F*/*F* in four directions as the response to one pair of temporal and spatial frequencies was taken, and the goodness (*R* ^2^) of a 2D elliptical Gaussian fitting as follows to evaluate the bias was used^6^

(2) $$\begin{array}{c} R\;\left(sf,tf\right)=Aexp\left(\frac{-{\left(\log_{2}sf-\log_{2}sf_{0}\right)}^{2}}{2{\left(\sigma_{sf}\right)}^{2}}\right)*\exp\left(\frac{-{\left(\log_{2}tf-\log_{2}tf_{p}\left(sf\right)\right)}^{2}}{2{\left(\sigma_{tf}\right)}^{2}}\right) \end{array}$$

where *A* is the peak response amplitude, *sf*_0_ and *tf*_0_ are the neuron's preferred spatial and temporal frequencies, respectively, and *σ*_sf_ and *σ*_tf_ are the tuning widths of spatial and temporal frequency, respectively. The dependence of temporal frequency preference on spatial frequency was captured by a power‐law exponent *ξ*, such that

(3) $$\begin{array}{c} \log_{2}\;\left(tf_{p}\left(sf\right)\right)=\xi \left(\log_{2}sf-\log_{2}sf_{0}\right)+\log_{2}tf_{0} \end{array}$$

### Slice Preparation, Confocal Imaging, and Analysis

To compare the labeling effect of the calcium probe on neuronal fibers, sectioning and confocal imaging of GCaMP6‐mRuby3.Membr labeled brain was performed. Mice were deeply anaesthetized with chloral hydrate and transcardially perfused with 0.01 m phosphate buffered saline (PBS, Sigma‐Aldrich Inc., St Louis, MO) and then 4% paraformaldehyde (PFA, Sigma‐Aldrich Inc., St Louis, MO). Brains were excised and postfixed in 4% PFA at 4 °C overnight. Coronal brain slices were cut to 100 µm thickness using a vibration microtome (Leica, VT120S). After a brief immersion in 0.05 m Na_2_CO_3_, the slices were mounted with 50% glycerin and imaged by a confocal microscope (Olympus FV3000, 20x/0.8).

SBR was calculated as the difference between foreground signal intensity (FSI) and background signal intensity (BSI) divided by BSI. A linear ROI (23 pixels) is drawn across a single fiber. The peak value is the FSI, and the average intensity of the four lowest pixels is the BSI.

The comparison of GCaMP6‐ with mRuby3.Membr‐labeling was quantified by calculating the proportion of colabeled axonal segments. Considering that the labeling pathways of these two proteins are different: GCaMP6 shows the neuronal morphology through cytosol, while mRuby3.Membr through cytomembrane, manual counting was chosen to allow for visual differences, such as strength, thickness, etc. The colabeling ratio was calculated by dividing the number of colabeling axonal segments to the number of mRuby3.Membr‐labeled segments.

### Whole‐Brain Imaging and Image Preprocessing

For whole‐brain imaging of the GCaMP6‐labeled mouse brain in vitro, a HD‐fMOST system^[^ ^18^ ^]^ that combines a line‐illumination modulation (LiMo) microscope with thin histological sectioning was used. All tissue preparation procedures have been previously described.^[^ ^51^, ^52^ ^]^ After in vivo calcium‐imaging, mouse brains were dissected and postfixed in 4% PFA for 24 h at 4 °C. The brains were rinsed in 0.01 m PBS (Sigma‐Aldrich) three times (for 2 h each) and embedded in Lowicryl HM20 resin (Electron Microscopy Sciences, 14 340). The embedded brains were imaged in a water bath containing propidium iodide (PI) and Na_2_CO_3_ (0.05 m) using the HD‐fMOST microscope at a voxel resolution of 0.325 × 0.325 × 1.0 µm^3^. There were two imaging channels, GCaMP6, and PI. The HD‐fMOST system automatically performed the cycles of imaging and sectioning until the brain‐wide data acquisition was complete. The raw data were saved at 16‐bit depth in LZW‐compression TIFF format.

Image preprocessing was performed using published methods.^[^ ^51^, ^53^ ^]^ Briefly, it included seamless mosaic stitching, image registration between the two channels, illumination correction and noise reduction for both GCaMP6 and PI channels. To facilitate data computing and analysis of the terabyte‐sized volume of data, the data format was transformed from TIFF to the native TDat format.

### Cross‐Modality Cell Matching

Using Amira Software (Version 6.1.1, FEI, Merignac Cedex, France) was used to identify and crop the soma region of labeled neurons around the injection site, then rotate the image block by referring to the cortical contour and take a rigid transformation to make its view similar to the two‐photon images. The blood vessels in the cranial window under a dissection microscope was photographed. The rough locations of calcium‐imaged somas can be narrowed down using the landmarks of blood vessels and the depth of two‐photon imaging. The maximum intensity projection (MIP) of an image block with appropriate thickness was used to compare to the functional image (template image mentioned in two‐photon image processing). The somas with distinct dendrites or blood vessel served as landmarks for affine transformation of functional images. The relationship of transformation between paired landmarks was

(4) $$R^{*},\;T^{*}={\mathrm{argmin}}_{R,T}∥P_{1}R^{T}+T-P_{2}∥$$

Where *T* is translation, *R* is a 2 × 2 matrix representing linear transformation, *P*_1_ is the coordinates of the landmarks in the functional image, and *P*_2_ is the is the coordinates of the landmarks in MIP. The number of landmarks is *n* (*n* > 2). Moore–Penrose (MP) inverse was used to resolve the relationship

(5) $$T^{*}=\mathrm{mean}\left(P_{2}\right)-\mathrm{mean}\left(P_{1}\right)$$

(6) $$R^{*}=P_{1}^{+}\left(\;P_{2}-T\right)$$

where $P_{1}^{+}$ is the MP inverse of *P*_1_. Then, the transformation matrix was applied to the functional image for manual matching. When there were less than three neurons in the functional image, affine transformation was skipped. Neurons were matched according to the patterns of fibers and the distribution of somas at the same time. It is recommended to capture 3D images during TPI to obtain more fiber information.

In the process above, all coordinate transformation parameters and soma coordinates were recorded. Finally, the coordinates of calcium‐imaged cells in the whole brain can be obtained through the inverse transform. The matching rate was calculated as the number of successfully matched neurons divided by the number of responsive somas in two‐photon images (the neurons that were sought to match) (Figure 2C).

### Whole‐Brain Neuron Reconstruction and Analysis

Neurons with visual responses were reconstructed in GTree software through human–computer interaction.^[^ ^54^ ^]^ Two skilled annotators independently worked on the reconstruction of each neuron, and then their consensual reconstructions were finally checked by a neuroanatomy expert. All reconstructed neurons were saved as SWC files. Then, they were mapped to the Allen Common Coordinate Framework (CCFv3)^[^ ^25^ ^]^ using the BrainsMapi methods.^[^ ^55^ ^]^

The tracing rate was calculated as dividing the number of neurons traced successfully by the number of neurons matched successful. The sample rate was quantified as dividing the number of neurons traced successfully by the number of neurons in in vivo images (≈500 µm^[^ ^2^ ^]^).

Neurons transmit information through their synaptic connections with downstream neurons. Considering the rich distribution of en‐passant boutons on the axon within the areas,^[^ ^56^, ^57^ ^]^ the axonal length in targets as the innervation density was quantified.^[^ ^1^ ^]^ At least 1.0 mm of axonal projection were required for an area to be considered as a target.^[^ ^2^, ^5^ ^]^

SNR measurements were performed according to procedures in a previous report.^[^ ^18^ ^]^ For the SNR of full‐length axons, the straight‐line distance from each node in the SWC file to soma (root node) was calculated and the nodes with an integer multiple of 40 µm were selected as sampling points. With each sampling point as the center, six neighborhood pixels were obtained and their mean pixel grayscale value was calculated as the average signal intensity. For axon endings, eight neighborhood pixels were obtained for calculation. SNR was calculated using the difference in average signal intensity with their surrounding background, divided by the standard deviation of background.

### Clustering

The K‐means clustering algorithm was used and subjects were grouped based on the Euclidean distances (Figure 4B). First centroids were chosen randomly. The data distribution and Calinski–Harabasz index were used to optimize the number of clusters (k), and *k* = 3.

### Statistical Analysis

Correlation analyses were performed using the nonparametric Spearman correlation test. They were considered as significant when *p*  <  0.05 and |*r*| >  0.6. Significant differences were determined by Kruskal–Wallis test with Dunn's post hoc test (Figure 5D–F; and Figure S11D–F, Supporting Information), Mann–Whitney test (Figure 4F), or Wilcoxon matched‐pairs signed rank test (Figure S6C, Supporting Information), two‐tailed. *p* < 0.05 (95% confidence interval) was considered statistically significant (ns, nonsignificant; *, *p* < 0.05; ****, *p* < 0.0001).

## Conflict of Interest

The authors declare no conflict of interest.

## Authors Contribution

W.Z. and S.K. contributed equally to this work. W.Z., S.K., P.L., Q.L., H.G., and W.S. conceived of and designed the study and wrote the manuscript. W.Z., S.K., Z.D., G.H., and Z.F. performed sparse labeling and in vivo two‐photon imaging. W.S. and Z.D. design the matrix stimulation protocol. W.Z., W.S., S.K., and Q.Z. calculated in vivo data from the matrix stimulation. W.Z. and S.K. analyzed functional projection data. H.G. and X.L. designed the tissue preparation. J.Y., R.J., T.J., and Q.L. conducted the in vitro whole brain imaging, A.L., W.L., X.J., L.S., and P.L. conducted image processing, cell matching and neuron reconstruction. W.Z., P.L., A.L., H.G., and Q.L. modified the manuscript.

## Supporting information

Supporting information

## Data Availability

The imaging data reported in the paper is available at http://atlas.brainsmatics.org/a/zhou2021. The custom code that supports the findings of this study is available from the corresponding author upon reasonable request.
