# Supplementary material for: Mapping the Function of Whole‐Brain Projection at the Single Neuron Level
Source: Adv Sci (Weinh). 2022 Oct 13;9(33):2202553. doi: 10.1002/advs.202202553 (PMC9685445; doi:10.1002/advs.202202553)
Supplement: Supplementary file 1 — Supporting information [file ADVS-9-2202553-s001.pdf]

ADVANCED SCIENCE
Open Access

Supporting Information

for *Adv. Sci.*, DOI 10.1002/advs.202202553

Mapping the Function of Whole-Brain Projection at the Single Neuron Level

Wei Zhou, Shanshan Ke, Wenwei Li, Jing Yuan, Xiangning Li, Rui Jin, Xueyan Jia, Tao Jiang, Zimin Dai, Guannan He, Zhiwei Fang, Liang Shi, Qi Zhang, Hui Gong, Qingming Luo, Wenzhi Sun*, Anan Li* and Pengcheng Li**

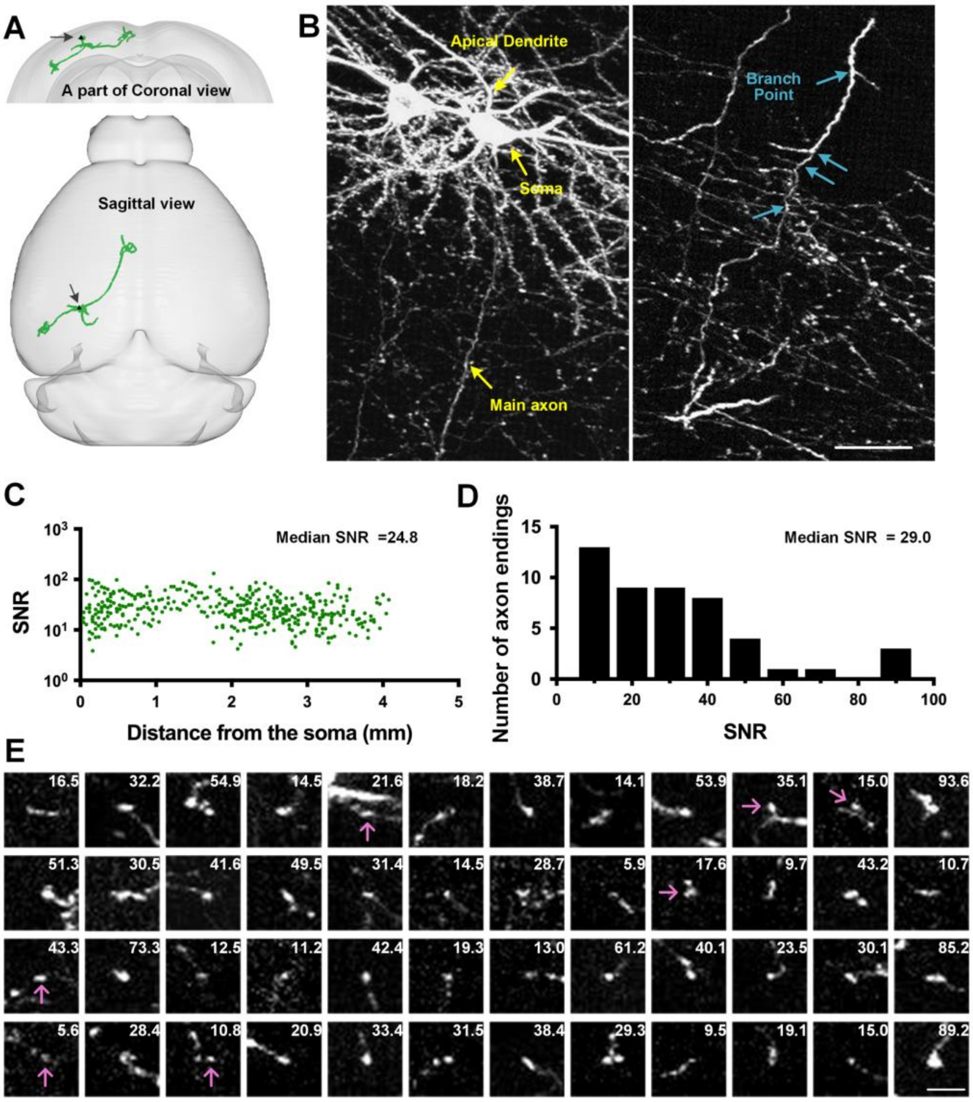

Figure S1. Brain-wide reconstruction of a single neuron labelled by GCaMP6. (**A**) Coronal view (top) and horizontal view (bottom) of Demo Neuron in **Fig. 1**. The soma is shown as a black triangle, and the green lines represent axons. The black arrows point to the locations of the insets shown in (**B**). (**B**) The yellow arrows indicate the apical dendrites, soma, and the main axon (left); and the blue arrows indicate all the branch points along the main axon (right). Scale bar, 20 µm. **(C)** Scatter plot of SNR for full-length axons. Median SNR = 24.8, quartile range: 15.7 ~ 40.1. The green dots represent the axonal SNR at different points that along the distances from soma. **(D)** Scatter plot of SNR for all axon endings shown in **(E)**, n = 48. Median SNR = 29.0, quartile range: 14.6 ~ 42.2. **(E)** Maximum-intensity projections (along the z axis) of all axon endings belonging to Demo Neuron. SNR is numbered in the upper right, and green arrows highlight the endings. Magenta arrows highlight the endings. Scale bar, 5 µm.

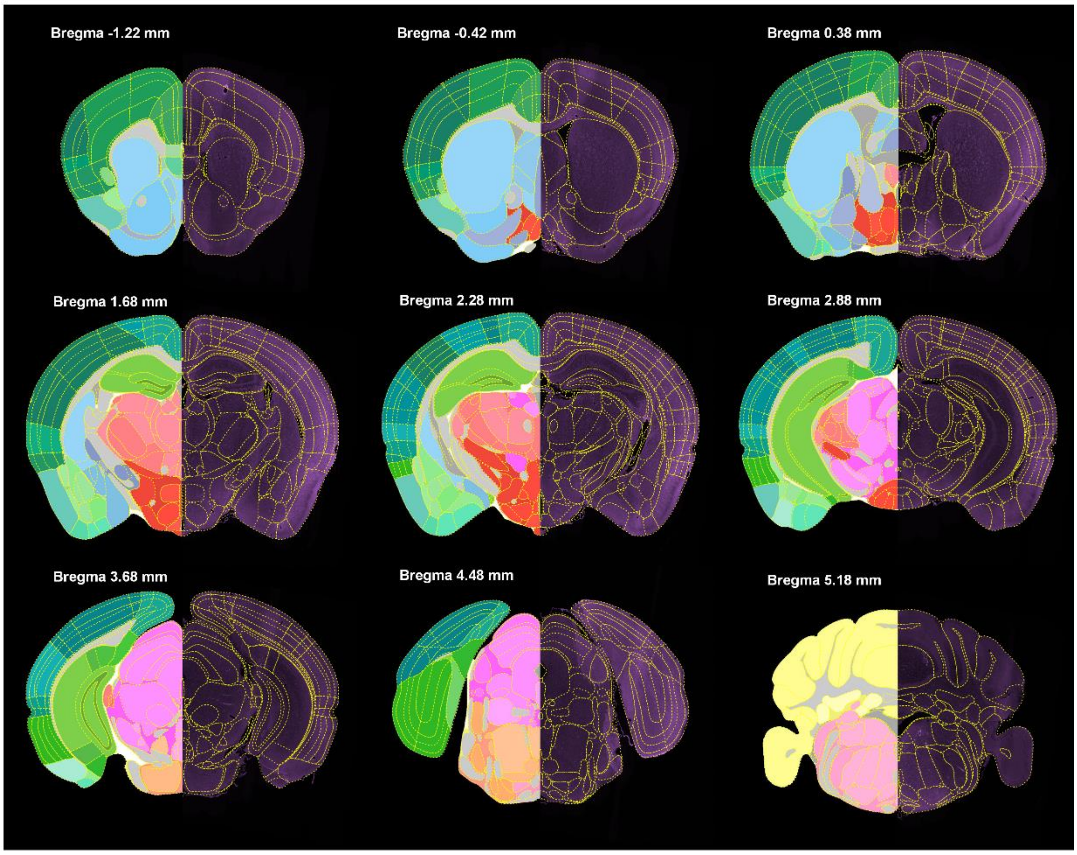

Figure S2. Registering fMOST with Allen CCFv3. Nine coronal sections of PI channel from fMOST dataset. Left subfigures: reference atlas from Allen CCFv3. Right subfigures: Allen CCFv3 (dotted line) superimposed on the registered fMOST images.

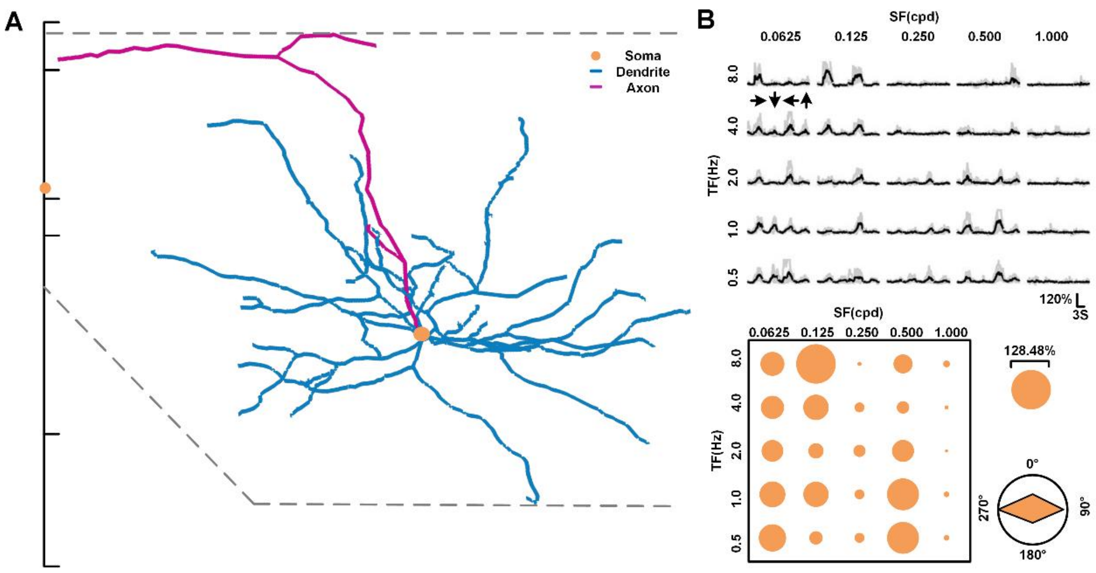

Figure S3. FAWPS of SST-Cre driven neuron. (**A**) Reconstruction of one SST-Cre driven neuron. The soma (yellow globe) is located in layer 2/3, dendrites (blue lines) are distributed in layers 2/3 and 4, and the axons (magenta lines) project to layer 1. (**B**) Functional tuning of the neuron shown in (**A**) to visual stimulation. Top: Calcium signal change evoked by the matrix visual stimulation. Bottom: Functional characteristics of the putative SST neuron. The maximum response is 128.48% (max ΔF/F), orientation selectivity is 0.35 (quantified by OSI), and spatiotemporal preference is 0.36 (quantified by R^2).

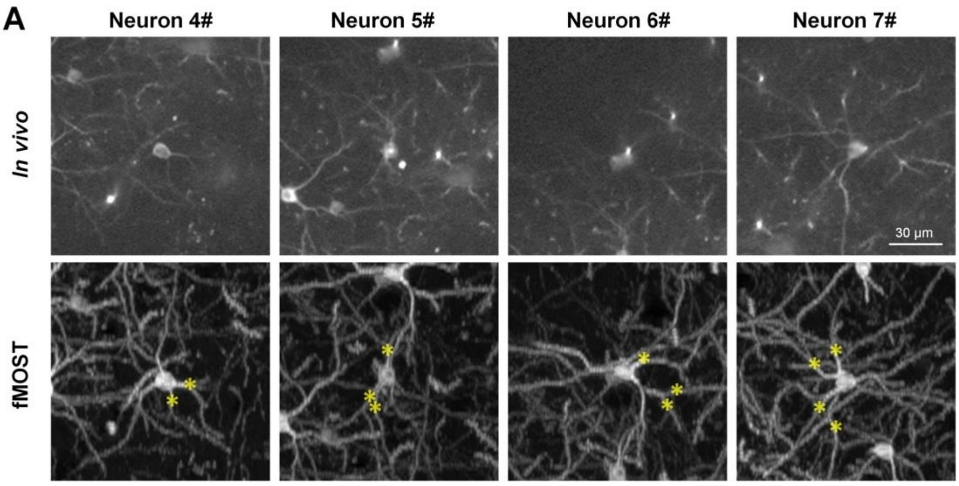

Figure S4. Cell matching of other neurons in Figure 2B. Top: calcium images obtained by two-photon imaging *in vivo*. Bottom: images obtained by HD-fMOST. The yellow asterisks identify the similar morphological features. Scale bar, 30 μm.

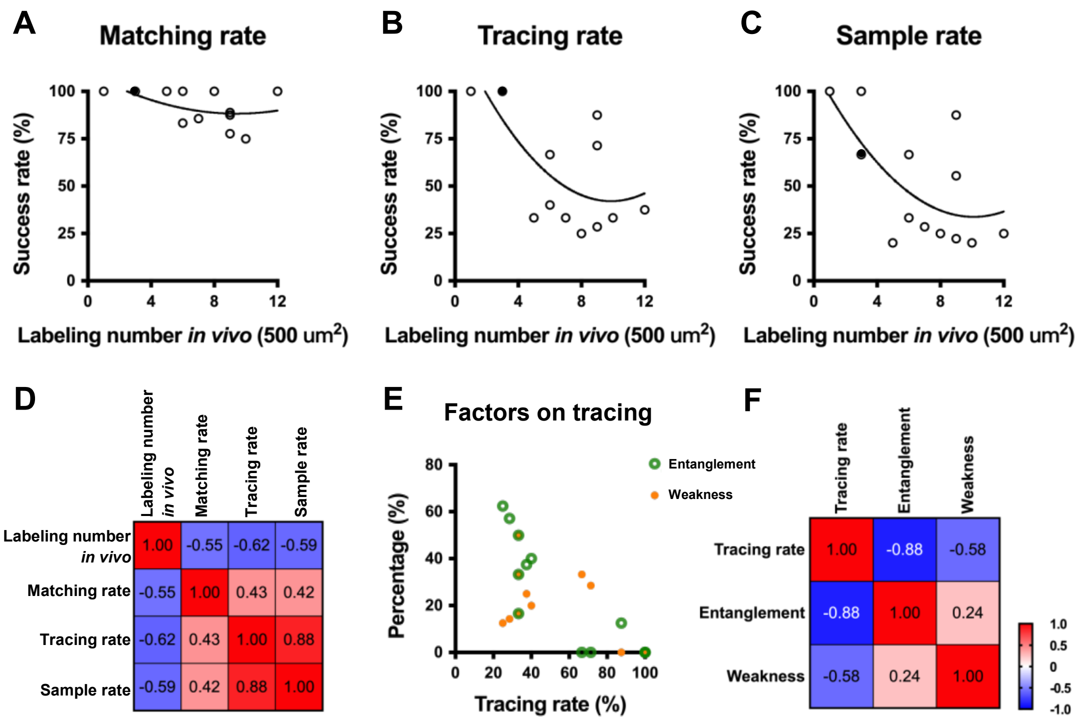

Figure S5. The effect of labeling density on the matching and tracing rates. (**A~C**) The matching rate, tracing rate and sampling rate were plotted as a function of the number of labeling cells *in vivo*, and fitted nonlinearly, respectively. Each circle represents n=1, the dots represent n = 3 in (**A~B)**, and n = 2 in (**C)**. (**D**) Correlation analysis of the cell number labeling *in vivo*, matching rate, tracing rate and sample rate. (**E**) The percentage of entanglement and weakness followed tracing rate. Note that four pieces of data overlap when tracing rate is 100%. (**F**) Correlation analysis of tracing rate, the percentage of entanglement and the percentage of weakness. Heatmap colors encode the degree of nonparametric *Spearman* Correlation. The correlation coefficients are indicated in heatmaps.

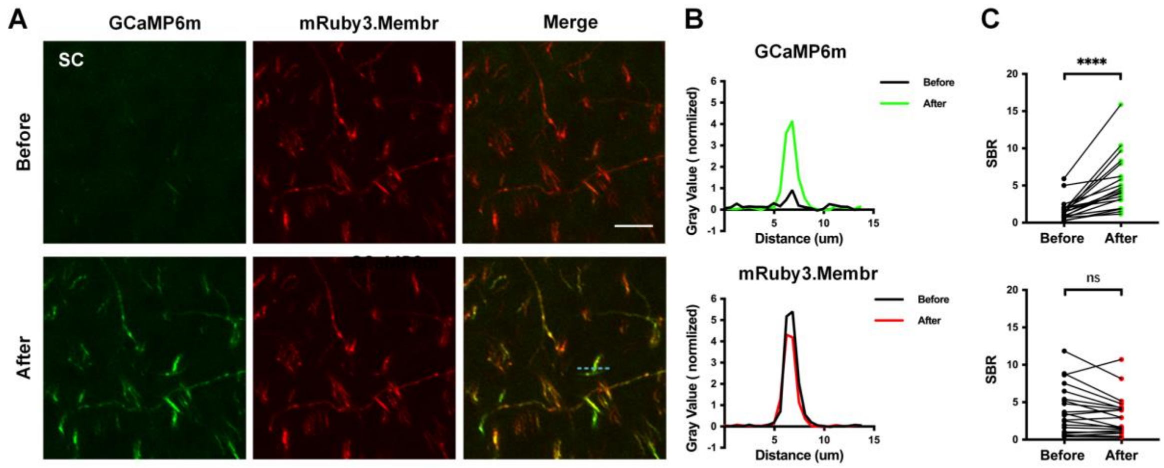

Figure S6. Enhancement of GCaMP6m signal by 0.05 M Na_2CO_3. (**A**) The example images of axons in SC before (top) and after (bottom) application of the alkaline buffer. Green, GCaMP6m; Red, mRuby3.Membr. Scar bar, 20 μm. (**B**) The line profile of GCaMP6m (top) and mRuby3.Membr (bottom) along the blue dashed line in (A), normalized by the background intensity. (**C**) The Signal-Background Ratio (SBR) of axons labeled by GCaMP6m (top) and mRuby3.Membr (bottom). Before, 4.07 ± 0.72; after, 3.27 ± 0.59. mean ± SEM, n = 20 axons from 2 mice. Wilcoxon matched-pairs signed rank test, two-tailed. ****P < 0.0001. ns, no significances (P > 0.05).

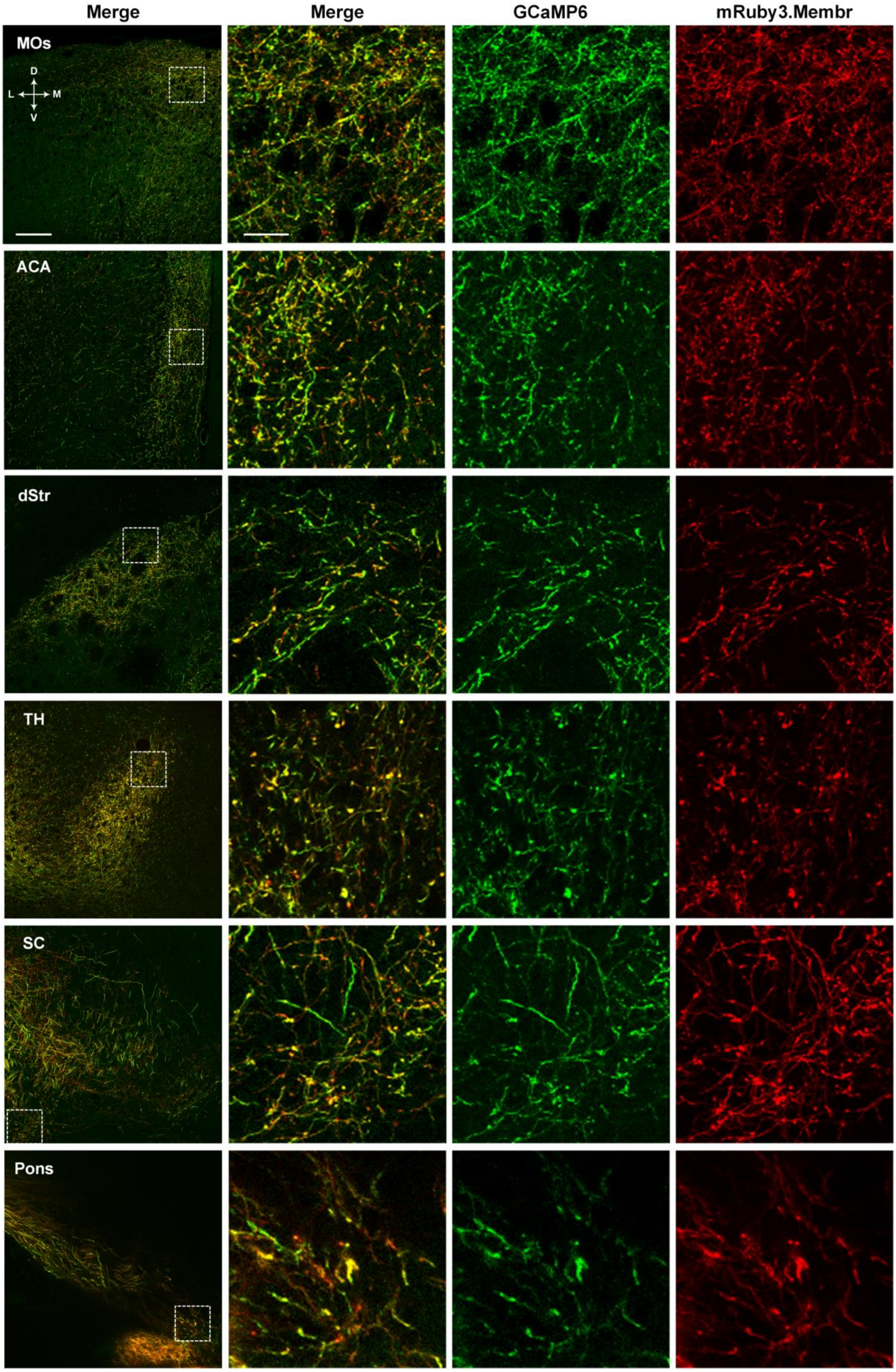
Merge
Merge
GCaMP6
mRuby3.Membr
MOs
D
L
M
V
ACA
dStr
TH
SC
Pons

Figure S7. The axons in target areas of VISp neurons co-labeled by GCaMP6m and mRuby3.Membr. A mixed virus solution containing AAV-hSyn-Cre, AAV-DIO-GCaMP6m and AAV-DIO-mRuby3.Membr (the concentration ratio was 1: 100: 100) was injected into VISp, and the axons in target areas were imaged using a confocal microscope after 2 weeks. The first column is a merge of red and green images. Scar bar, 100 μm. The right three columns are enlarged from the box in the first column. Scar bar, 20 μm. Green, GCaMP6m; Red, mRuby3.Membr. ACA, Anterior cingulate area; dStr, dorsal-Striatum; TH, Thalamus; SC, Superior Colliculus.

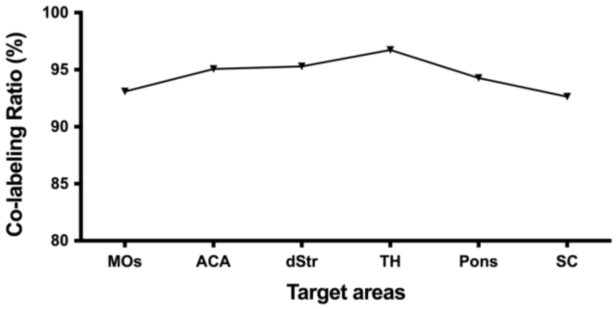

Figure S8. The co-labeling ratio of axonal segments labeled by GCaMP6m and mRuby3.Membr. The co-labeling ratio is showed as the percentage of co-labeled axonal segments in mRuby3.Membr-labeled axonal segments, and the image data are from the first column in **Figure S7**. The co-labeling ratio in these six target areas ranged from 92.64% to 96.74%, and their average value is 94.52% ± 1.52% (mean ± SD).

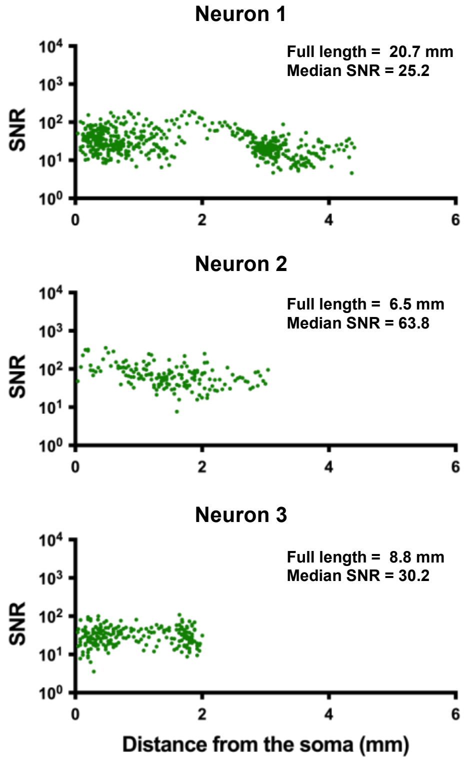

Figure S9. SNR values at different points along the intact axons of three reconstructed neurons. SNRs for intact axons in the individual neurons, which are indicated in **Figure. 3B**. The green dots represent the axonal SNRs at different points that along the distance from soma. The full length and median SNR of axons are indicated in the upper right corner.

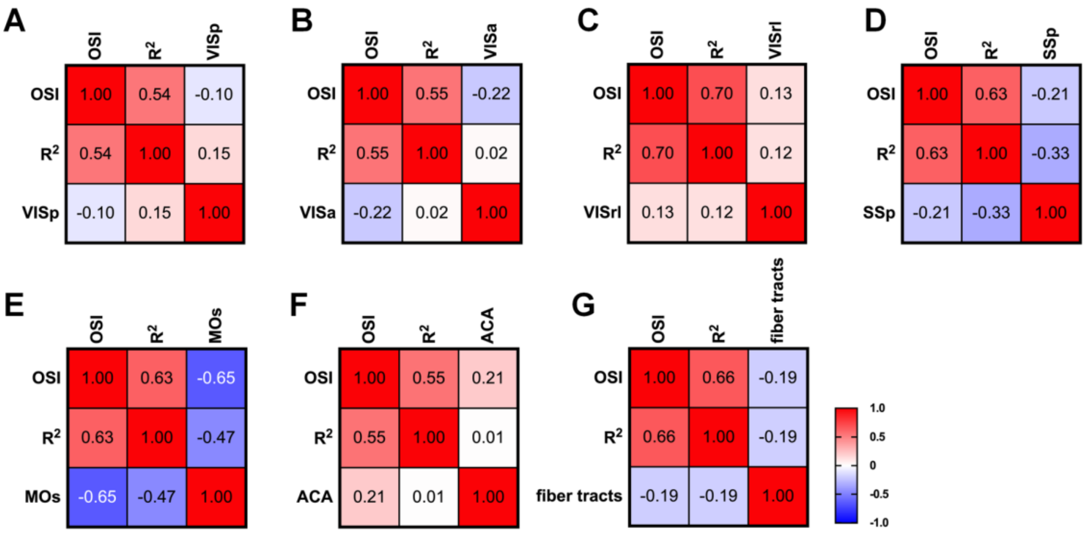

Figure S10. Correlation analysis of axonal length and functional characteristics in seven regions. (A) VISp-projecting neurons, n=24. (**B**) VISa-projecting neurons, n=23. (**C**) VISrl-projecting neurons, n=31. (**D**) SSp-projecting neurons, n=19. (**E**) MOs-projecting neurons, n=17. (**F**) ACA-projecting neurons, n=10. (**G**) fiber tracts-projecting neurons, n=17. Heatmap colors encode the degree of nonparametric Spearman correlation.

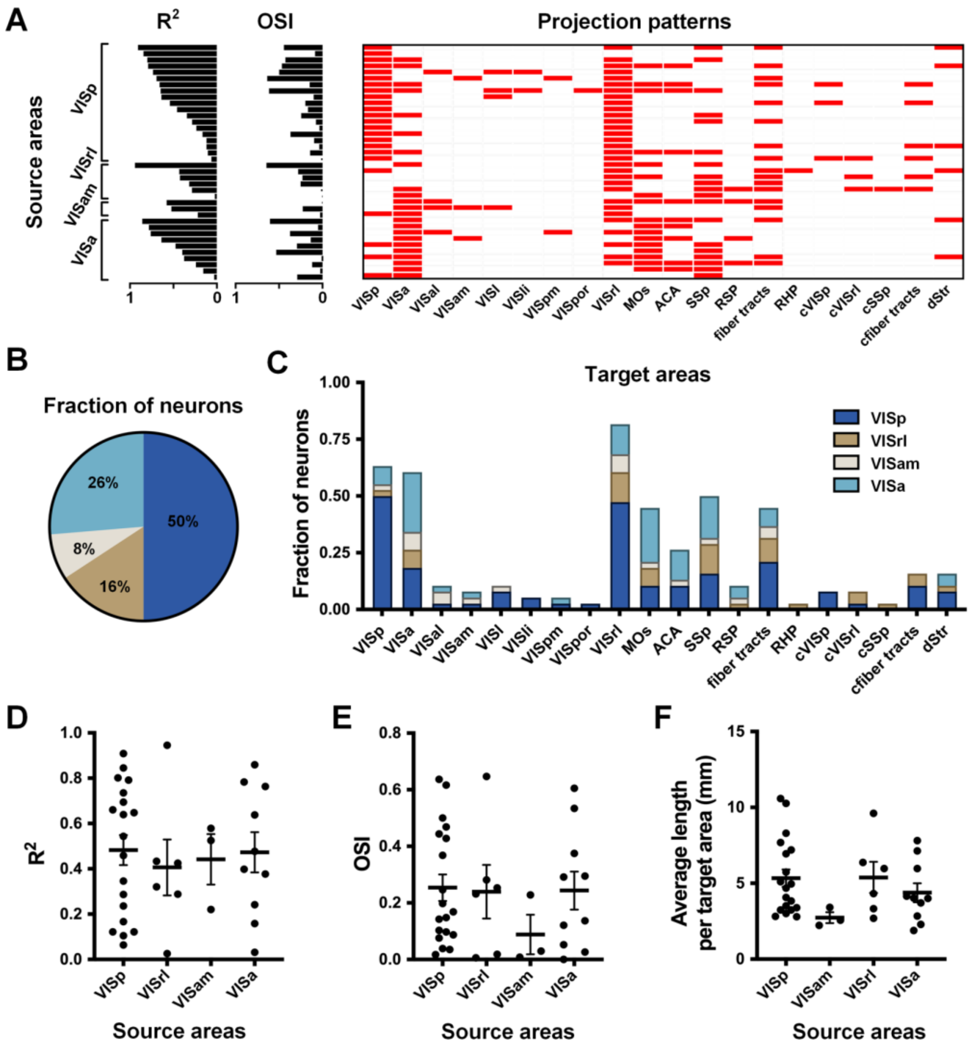

Figure S11. FAWPS of L2/3 neurons from different subregions of visual cortex. (**A**) The FAWPS of neurons located in VISp (n = 19), VISrl (n = 6), VISam (n = 3), and VISa (n = 10). Rows show individual neurons, and red indicated the targeted areas. (**B**) The fraction of neurons from different source areas. VISp, 50%; VISrl, 16%; VISam, 8%; VISa, 26%. (**C**) The fraction of neurons in target areas from different source areas. Colors represent the source areas. (**D**～**F**) Scatter plots of R^2, OSI and the average length of single target area. Error bars, ± SEM. Kruskal–Wallis test with Dunn's post hoc test, and there are no significances ($P > 0.05$).
